# Protein tyrosine phosphatase receptor type O (PTPRO) knockdown enhances the proliferative, invasive and angiogenic activities of trophoblast cells by suppressing ER resident protein 44 (ERp44) expression in preeclampsia

**DOI:** 10.1080/21655979.2021.1997561

**Published:** 2021-12-11

**Authors:** Yang Yang, Xiaoxia Qiu, Fang Wang

**Affiliations:** a Department of Obstetrics and Gynecology, Shulan (Hangzhou) Hospital Affiliated to Zhejiang Shuren University Shulan International Medical College, Hangzhou, Zhejiang, P.R. China; b Department of Obstetrics and Gynecology, The Affiliated Shanghai East Hospital, Tongji University, Shanghai, P.R. China

**Keywords:** PTPRO, ERp44, trophoblast cells, preeclampsia

## Abstract

Preeclampsia (PE), a pregnancy-specific syndrome, is the primary cause of maternal mortality. This work was designed to investigate the specific functions of PTPRO/ ERp44 in the biological behaviors of trophoblast cells and elucidate the underlying molecular mechanism. Constructed siRNA-PTPRO and ERp44 overexpression plasmids were transfected into HTR-8/SVneo and JEG-3 cells for further functional experiments. Subsequently, the proliferation and invasion of trophoblast cells were identified by performing CCK-8, flow cytometry and transwell assay. In addition, tube formation assay was employed to estimate the angiogenic ability of HUVECs incubated with the conditioned media (CM) of HTR-8/SVneo or JEG-3 cells. Importantly, the interaction between PTPRO and ERp44 was analyzed through Co-IP. In the current investigation, it was discovered that downregulation of PTPRO notably facilitated the proliferation and invasion of trophoblast cells and induced a stronger in vitro angiogenesis. Moreover, PTPRO interacted with ERp44 to regulate ERp44 expression. ERp44 overexpression suppressed the proliferative, invasive and angiogenic activities of trophoblast cells. As a result, functions of PTPRO knockdown in the biological behaviors of trophoblast cells were partially abrogated upon elevation of ERp44. To sum up, this current research systematically evidenced that PTPRO could regulate the biological behaviors of trophoblast cells by modulating ERp44. Findings may contribute to a novel therapeutic strategy for PE.

## Introduction

Preeclampsia (PE) is one of the hypertensive disorders during pregnancy [1]. It has been proved that PE greatly contributes to the increasing maternal and perinatal morbidity and mortality around the world. Nevertheless, present studies fail to find out its precise etiology and pathogenesis [2,3]. Placental dysfunction is a key characteristic of PE, which is closely associated with abnormal biological function of trophoblasts (proliferation, migration, invasion and apoptosis) [4]. So, it is urgent to explore the molecular mechanisms underlying inadequate trophoblastic invasion and growth, aiming to accurately understand the pathogenesis of PE.

Protein tyrosine phosphatase receptor type O (PTPRO) is a novel protein tyrosine phosphatase [5]. It consists of a transmembrane region, a single intracellular catalytic domain and an extended extracellular domain [6]. PTPRO participates in various cellular processes, including proliferation, apoptosis, migration, differentiation and communication [7,8]. Importantly, PTPRO has been reported to be upregulated in placental mononuclear cells of PE patients [9]. However, the functional role of PTPRO in the physiology and disorders of placental trophoblasts has not been fully understood till now.

We analyzed the PTPRO gene network using the Biological General Repository for Interaction Datasets (BioGRID: http://thebiogrid.org). PTPRO can be highly combined with the ER resident protein 44 (ERp44). ERp44 is a member of the protein disulfide isomerase (PDI) family [10]. ERp44 is not only a critical player in the process of mediating the localization of key enzymes, but also a regulator that controls the traffic and oligomeric assembly of disulfide-linked oligomeric proteins [11,12]. Apart from the facts mentioned above, it is worth noting that ERp44 influences PE progression in diverse ways. Zou et al. reported that rises of ERp44 appeared in placental tissues of PE. Meanwhile, the proliferation and invasion of trophoblast cells were suppressed by ERp44 overexpression. In this process, angiogenesis of HUVEC cells was inhibited as well [13]. Besides, downregulation of ERp44 has been reported to arrest the apoptosis of trophoblast cells and repress ER stress-induced apoptotic proteins [14].

This work explored the involvement of PTPRO and ERp44 in trophoblast cells from a biological perspective. Additionally, a proteomic analysis was carried out to identify the interaction between PTPRO and ERp44. What’s more, the molecular mechanisms underlying the participation of PTPRO and ERp44 in the development of PE were elucidated in vitro.

## Materials and methods

### Cell culture

American Type Culture Collection (ATCC, VA, USA) was the supplier of trophoblast cell lines (HTR-8/SVneo and JEG-3) and HUVECs. HTR-8/SVneo cells and HUVECs were cultured in Roswell Park Memorial Institute 1640 (RPMI-1640; Gibco, NY, USA) containing 10% fetal bovine serum (FBS; Gibco, NY, USA) and 1% penicillin/streptomycin. JEG-3 cells were cultured in Dulbecco’s modified Eagle’s medium/Nutrient Mixture F-12 (DMEM/F12; Gibco, NY, USA) containing 10% FBS and 1% penicillin/streptomycin. Cells were maintained in a humid incubator at 37°C with 5% CO_2_.

### Cell transfection

PTPRO small interfering RNA (siRNA-PTPRO) and the negative control (siRNA-NC) were constructed from GenePharma Company (Shanghai, China). The ERp44 overexpression plasmid (Ov-ERp44) was designed and synthesized by inserting ERp44 cDNA fragment into the pcDNA3.1 vector (GenePharma, Shanghai, China) and the empty pcDNA3.1 vector served as the negative control (Ov-NC). Cell transfection was performed using Lipofectamine 2000 (Invitrogen, CA, USA) according to the manufacturer’s instructions.

### Cell counting kit (CCK)-8 assay

CCK-8 assay was employed to measure cell viability. Briefly, HTR8/SVneo or JEG-3 cells (2,000/well) were seeded into 96-well plates. 24, 48 or 72 h post incubation, 10 µl CCK-8 reagent (Beyotime, Shanghai, China) was added into each well and incubated for 4 h. Then, the optical value (OD) (at 450 nm) was measured using a microplate reader (Bio-Rad, CA, USA).

### Cell cycle analysis

Cell cycles of HTR-8/SVneo and JEG-3 cells were detected through flow cytometry. In brief, cells were harvested and fixed with 70% ethanol at −4°C for 3 days. Next, cells were stained using a mixture of PI (10 mg/mL), RNase (100 mg/mL) and PBS for 30 min at room temperature. The proportions of cells in different stages of cell cycle were analyzed by a flow cytometer (BD Biosciences, CA, USA)

### Cell invasion assay

Transwell assay was performed to assess cell invasion. Cells were collected and resuspended in serum-free medium and seeded onto the transwell chambers (Corning, NY, USA) precoated with Matrigel (BD Biosciences, CA, USA) at a density of 2 × 10^4^ cells/well. Next, 600 µl medium containing 10% FBS was placed to the lower chamber. After 24 h incubation, cells on the lower layer were fixed with methanol and stained by crystal violet (Solarbio, Beijing, China). The number of penetrated cells was counted under a microscope (Leica, Wetzlar, Germany).

### Tube formation assay

In brief, the conditioned media (CM) of HTR-8/SVneo and JEG-3 cells were collected. HUVECs were seeded on 96-well plates precoated with 50 μl Matrigel (BD Biosciences, CA, USA) at a density of 2 × 10^4^ cells/well. Then, HUVECs were incubated with 250 µl CM for 24 h. An inverted microscope (Olympus, Tokyo, Japan) was employed to observe the tube formation.

### Western blot analysis

RIPA lysis buffer (Beyotime, Shanghai, China) was applied to extract protein and the protein concentration was estimated using bicinchoninic acid method. Protein extracts were separated by SDS-PAGE and then transferred to PVDF membranes (Beyotime, Shanghai, China). Membranes were incubated with primary antibodies against PTPRO (Abcam, ab231560, 1:1000), MMP2 (Abcam, ab181286, 1:1000), MMP9 (Abcam, ab228402, 1:1000), Ki67 (Abcam, ab16667, 1:1000), VEGF (Abcam, ab214424, 1:1000), ET-1 (Abcam, ab2786, 1:1000), sFIt-1 (Abcam, ab32152, 1:1000), ERp44 (Abcam, ab137611, 1:3000) and GAPDH (Abcam, ab181602, 1:10,000) overnight at 4°C. On the second day, membranes were exposed to a horseradish peroxidase (HRP)-labeled secondary antibody at room temperature for 1 h. Protein signals were developed with an enhanced chemiluminescence (ECL) detection kit (Beyotime, Shanghai, China). Protein expression was analyzed using Image J software with GAPDH as a loading control.

### Reverse transcription quantitative polymerase chain reaction (RT-qPCR)

TRIzol reagent (Takara, Dalian, China) was used to extract total RNA. Total RNA concentration and purity were determined by a NanoDrop ND-1000 spectrophotometer (Thermo Fisher Scientific, DE, USA). Next, 1 µg RNA was reversely transcribed into cDNA using a PrimeScript RT kit (Takara, Tokyo, Japan). Levels of mRNAs were measured using SYBR Master Mixture (Takara, Dalian, China) and calculated with 2^–ΔΔCt^ method [15]. The sequences of the primers were as follows: PTPRO forward: 5′- TATTGTGAGCCTCCGTGTGT −3′, reverse: 5′- GCCAAGCCTTTTCAGTGACA −3′; ERp44 forward: 5′-CCTGTGCCAGGCCTCAATAC −3′, reverse: 5′-TGGCACTGGGCTTCCTGATA −3′; MMP-2 forward: 5′-GCCCCAGACAGGTGATCTTG-3′, reverse, 5′-GCTTGCGAGGGAAGAAGTTGT-3′; MMP-9 forward: 5′- AGACGGGTATCCCTTCGACG −3′, reverse, 5′-AAACCGAGTTGGAACCACGAC −3′; GAPDH forward: 5′- ACCACAGTCCATGCCATCAC −3′, reverse, 5′- TCCACCACCCTGTTGCTGTA −3′.

### Co-immunoprecipitation (Co-IP)

Co-IP assay was employed to analyze the interaction between PTPRO and ERp44. Cells were lysed using IP lysis buffer (Beyotime, Shanghai, China). Specific antibodies or IgG were added into the supernatant of cell lysates and incubated overnight at 4°C. The subsequent incubation of complexes was done with protein A/G beads (Santa Cruz, CA, USA) for 2 h. At last, the Co-IP products were harvested after being washed 3 times with lysis buffer and analyzed by western blotting.

### Statistical analysis

Data were expressed as mean values ± SD. One-way analysis of variance (ANOVA) followed by Tukey’s post hoc test was adopted to analyze the differences among different groups of participants. P-values less than 0.05 indicated differences with statistical significance.

## Results

### Downregulation of PTPRO promoted the proliferation of trophoblast cells

**Figure 1.**
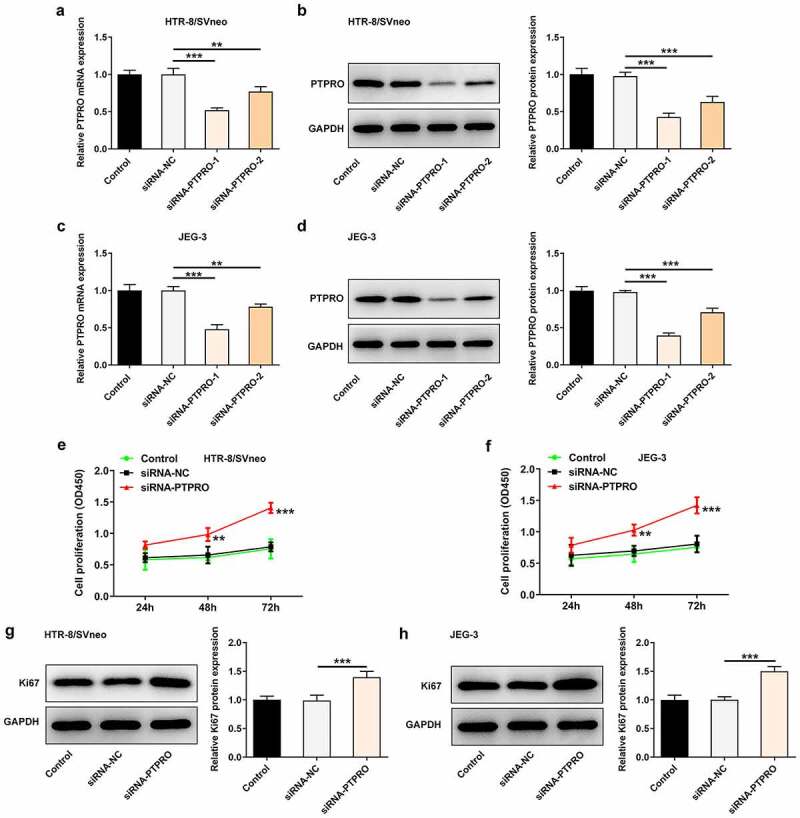
Downregulation of PTPRO promoted the proliferation of trophoblast cells. HTR-8/SVneo and JEG-3 cells were transfected with siRNA-PTPRO-1/2 or siRNA-NC. (a) RT-qPCR was employed to detect the mRNA level of PTPRO in HTR-8/SVneo cells. (b) Western blot analysis was employed to detect the protein level of PTPRO in HTR-8/SVneo cells. (c) RT-qPCR was employed to detect the mRNA level of PTPRO in JEG-3 cells. (d) Western blot analysis was employed to detect the protein level of PTPRO in JEG-3 cells. (e) CCK-8 assay was employed to assess the proliferation of HTR-8/SVneo cells. (f) CCK-8 assay was employed to assess the proliferation of JEG-3 cells. (g) Western blot analysis was employed to detect the protein level of Ki67 in HTR-8/SVneo cells. (h) Western blot analysis was employed to detect the protein level of Ki67 in JEG-3 cells. **p < 0.01, ***p < 0.001

In order to identify the specific functions of PTPRO in the biological behaviors of trophoblast cells, siRNA-PTPRO-1 or siRNA-PTPRO-2 were introduced into HTR-8/SVneo and JEG-3 cells. The transfection efficiency was validated by performing RT-qPCR and western blotting analysis. The mRNA and protein levels of PTPRO markedly decreased after transfection (Figure 1(a-d)). Due to the optimized transfection efficiency, siRNA-PTPRO-1 was selected for subsequent experiments. Then, results of CCK8 assays revealed that downregulation of PTPRO significantly enhanced the proliferative abilities of HTR-8/SVneo and JEG-3 cells (Figure 1(e, f)). Additionally, elevated expressions of Ki67 in HTR-8/SVneo and JEG-3 cells transfected with siRNA-PTPRO-1 further confirm the enhancement of PTPRO knockdown on the proliferation of trophoblast cells (Figure 1(g, h)).

### Downregulation of PTPRO promoted cell cycle progression

**Figure 2.**
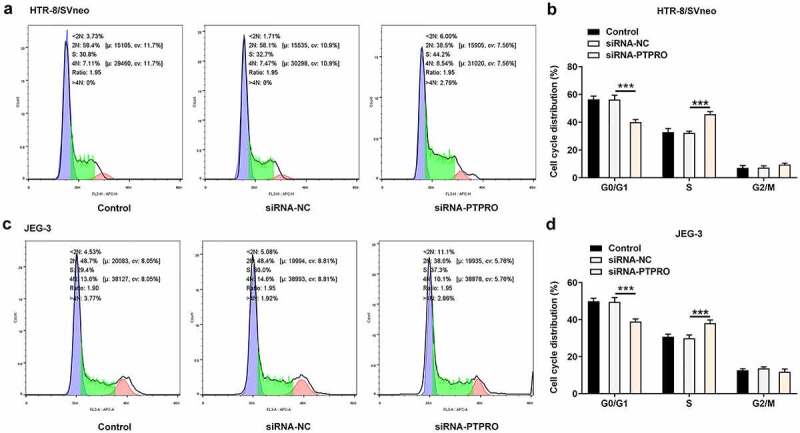
Downregulation of PTPRO promoted cell cycle progression. HTR-8/SVneo and JEG-3 cells were transfected with siRNA-PTPRO or siRNA-NC. (a, b) Flow cytometry was adopted to evaluate the cell cycle distribution of HTR-8/SVneo cells. (c, d) Flow cytometry was adopted to evaluate the cell cycle distribution of JEG-3 cells. ***p < 0.001

According to the CCK8 results, cell cycle distribution was evaluated by flow cytometry. In both HTR-8/SVneo and JEG-3 cells, transfection with siRNA-PTPRO resulted in a decrease in the proportion of cells in G0/G1 phase and an increase in the proportion of cells in S phase, indicating that the cell cycle was facilitated during the G0/G1 to S phase transformation by PTPRO knockdown (Figure 2(a-d)).

### Downregulation of PTPRO facilitated the invasion of trophoblast cells

**Figure 3.**
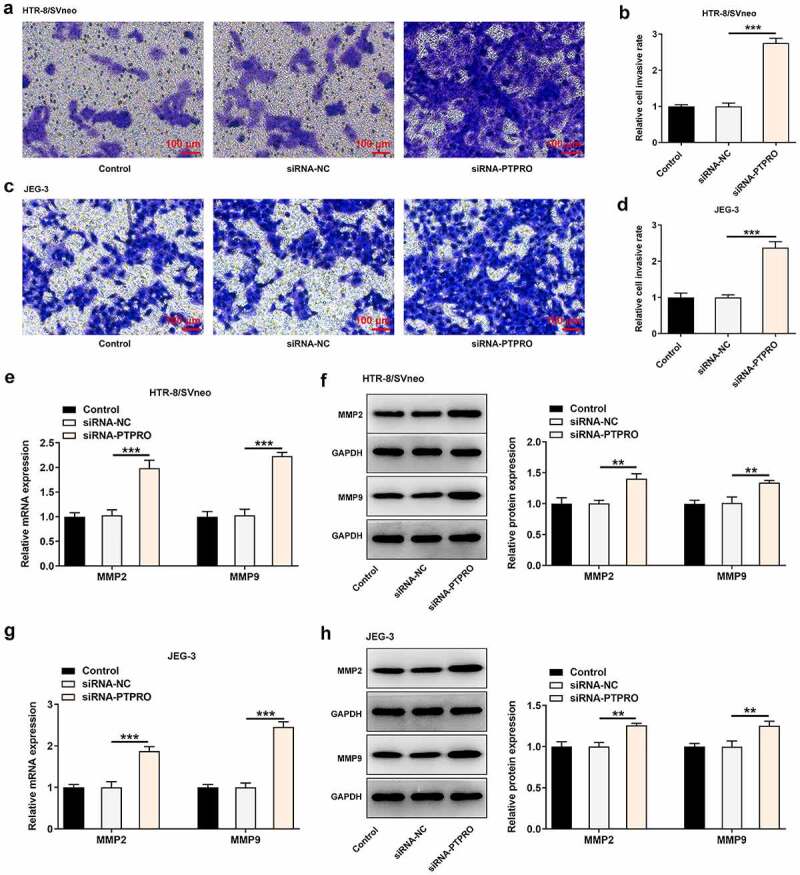
Downregulation of PTPRO facilitated the invasion of trophoblast cells. HTR-8/SVneo and JEG-3 cells were transfected with siRNA-PTPRO or siRNA-NC. (a, b) Transwell assay was performed to evaluate the invasion of HTR-8/SVneo cells. (c, d) Transwell assay was performed to evaluate the invasion of JEG-3 cells. (e) RT-qPCR was employed to detect the mRNA levels of MMP2 and MMP9 in HTR-8/SVneo cells. (f) Western blot analysis was employed to detect the protein levels of MMP2 and MMP9 in HTR-8/SVneo cells. (g) RT-qPCR was employed to detect the mRNA levels of MMP2 and MMP9 in JEG-3 cells. (h) Western blot analysis was employed to detect the protein levels of MMP2 and MMP9 in JEG-3 cells. **p < 0.01, ***p < 0.001

Transwell assay was essential in evaluating the invasive abilities of trophoblast cells. It was observed that downregulation of PTPRO enhanced the invasive abilities of HTR-8/SVneo and JEG-3 cells (Figure 3(a-d)). Besides, it was testified that MMP2 and MMP9 in HTR-8/SVneo and JEG-3 cells were obviously increased upon knockdown of PTPRO (Figure 3(e-h)). Results above collectively supported that PTPRO knockdown notably facilitated the invasion of trophoblast cells.

### Downregulation of PTPRO induced a stronger in vitro angiogenesis

**Figure 4.**
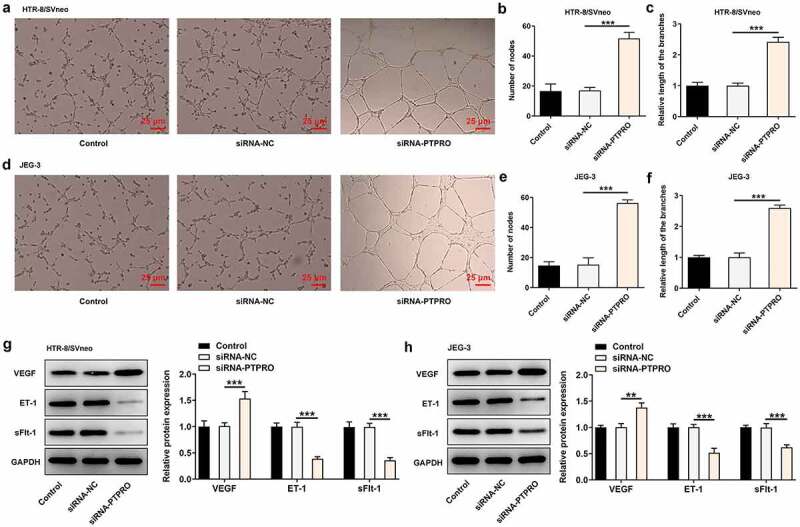
Downregulation of PTPRO induced a stronger in vitro angiogenesis. HTR-8/SVneo and JEG-3 cells were transfected with siRNA-PTPRO or siRNA-NC. HUVECs were incubated with the conditioned media (CM) of HTR-8/SVneo or JEG-3 cells. (a-c) The angiogenic activity of HUVECs incubated with the conditioned media (CM) of HTR-8/SVneo cells was tested using an in vitro tube formation assay. (d-f) The angiogenic activity of HUVECs incubated with the conditioned media (CM) of JEG-3 cells was tested using an in vitro tube formation assay. (g) Western blot analysis was employed to detect the protein levels of VEGF, ET-1 and sFlt-1 in HUVECs incubated with the conditioned media (CM) of HTR-8/SVneo cells. (h) Western blot analysis was employed to detect the protein levels of VEGF, ET-1 and sFlt-1 in HUVECs incubated with the conditioned media (CM) of JEG-3 cells. **p < 0.01, ***p < 0.001

The angiogenic activity was tested via an in vitro tube formation assay. It was discovered that in vitro angiogenesis was facilitated upon PTPRO knockdown (Figure 4(a-f)). In addition, strikingly increased expression of VEGF and markedly decreased expressions of ET-1 and sFlt-1 caused by knockdown of PTPRO together evidenced that PTPRO knockdown induced a stronger in vitro angiogenesis (Figure 4(g, h)).

### PTPRO interacted with ERp44 to participate in PE progression

**Figure 5.**
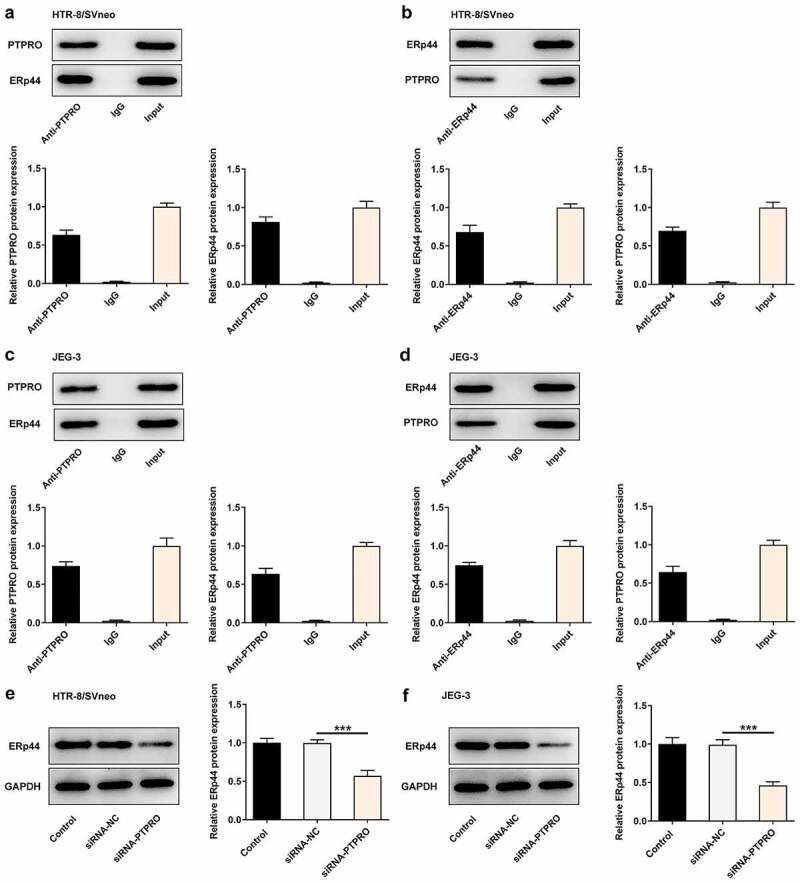
PTPRO interacted with ERp44 to participate in PE progression. (a-d) Co-IP assays were carried out to determine the interaction between PTPRO and ERp44. (e, f) HTR-8/SVneo and JEG-3 cells were transfected with siRNA-PTPRO or siRNA-NC. Western blot analysis was employed to detect the protein level of ERp44 in HTR-8/SVneo and JEG-3 cells. ***p < 0.001

To clarify the molecular mechanism underlying PTPRO in PE, we analyzed PTPRO gene network using the Biological General Repository for Interaction Datasets (BioGRID: http://thebiogrid.org). It was displayed that PTPRO could highly combine with ERp44. Additionally, the direct interaction between PTPRO and ERp44 in HTR-8/SVneo and JEG-3 cells was confirmed by performing Co-IP assays. Co-IP assay presented that ERp44 existed in anti-PTPRO group, indicating that PTPRO physically interacted with ERp44 (Figure 5(a, c)). Meanwhile, the interaction between PTPRO and ERp44 was inversely confirmed by Co-IP assay in HTR-8/SVneo and JEG-3 cells. PTPRO existed in anti-ERp44 group (Figure 5(b, d)). Furthermore, it was verified that knockdown of PTPRO downregulated ERp44 expression in trophoblast cells (Figure 5(e, f)).

### Downregulation of PTPRO promoted the proliferation of trophoblast cells by suppressing ERp44 expression

**Figure 6.**
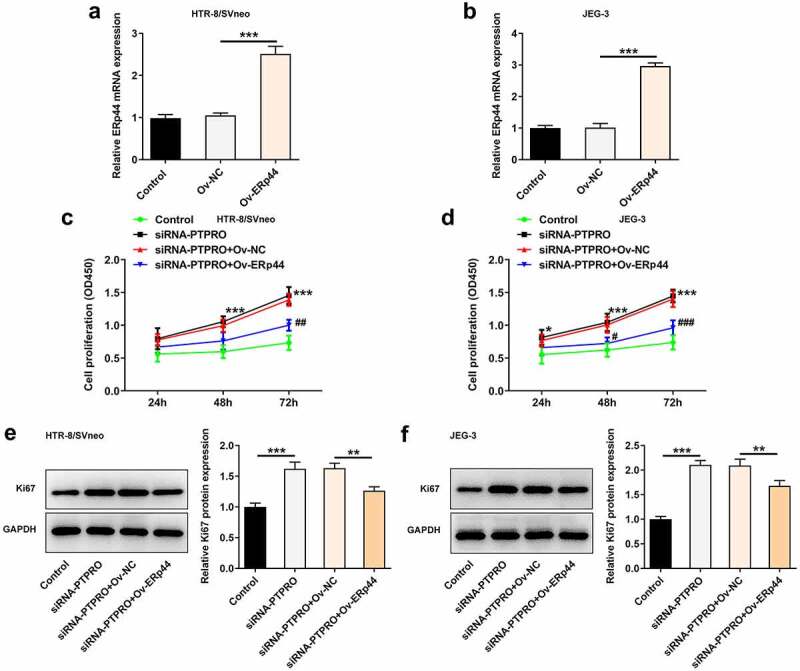
Downregulation of PTPRO promoted the proliferation of trophoblast cells by suppressing ERp44 expression. (a, b) HTR-8/SVneo and JEG-3 cells were transfected with Ov-ERp44 or Ov-NC. RT-qPCR was employed to detect the mRNA level of ERp44 in HTR-8/SVneo and JEG-3 cells. (c, d) HTR-8/SVneo and JEG-3 cells were transfected with siRNA-PTPRO or co-transfected with siRNA-PTPRO and Ov-ERp44. CCK-8 assay was employed to assess the proliferation of HTR-8/SVneo and JEG-3 cells. (e, f) HTR-8/SVneo and JEG-3 cells were transfected with siRNA-PTPRO or co-transfected with siRNA-PTPRO and Ov-ERp44. Western blot analysis was employed to detect the protein level of Ki67 in HTR-8/SVneo and JEG-3 cells. *p < 0.05, **p < 0.01, ***p < 0.001; ^#^p < 0.05, ^##^p < 0.01, ^###^p < 0.001

ERp44 overexpression plasmids were transfected into trophoblast cells to upregulate ERp44 expression, aiming to identify the precise functions of ERp44 in the biological behaviors of trophoblast cells and probe into the molecular mechanism underlying PTPRO in PE. Transfection with Ov-ERp44 distinctly elevated ERp44 expression in HTR-8/SVneo and JEG-3 cells (Figure 6(a, b)). Results of CCK-8 assay and western blotting analysis suggested that the enhancement of PTPRO knockdown on the proliferation of trophoblast cells was partly reversed upon elevation of ERp44 (Figure 6(c-f)).

### Downregulation of PTPRO promoted cell cycle progression by suppressing ERp44 expression

**Figure 7.**
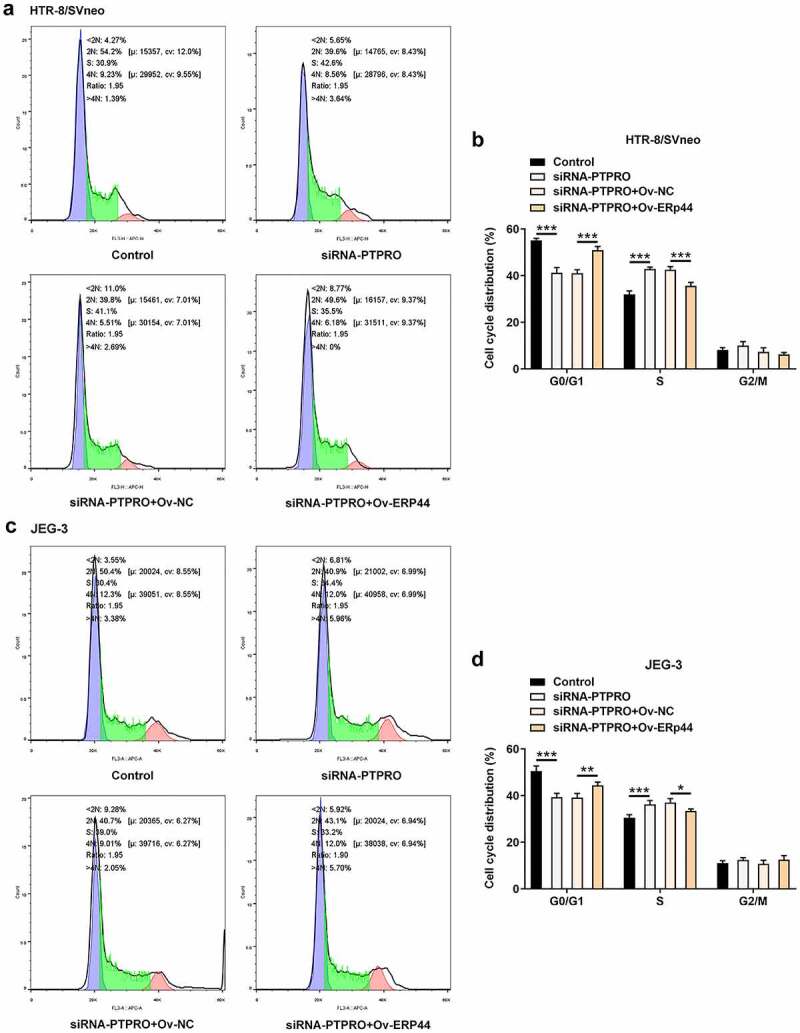
Downregulation of PTPRO promoted cell cycle progression by suppressing ERp44 expression. HTR-8/SVneo and JEG-3 cells were transfected with siRNA-PTPRO or co-transfected with siRNA-PTPRO and Ov-ERp44. (a, b) Flow cytometry was adopted to evaluate the cell cycle distribution of HTR-8/SVneo cells. (c, d) Flow cytometry was adopted to evaluate the cell cycle distribution of JEG-3 cells. *p < 0.05, **p < 0.01, ***p < 0.001

In this work, it had been verified that PTPRO knockdown facilitated cell cycle. Then, upregulation of ERp44 elevated the proportion of cells in G0/G1 phase and reduced the proportion of cells in S phase, boosting cell cycle arrest in HTR-8/SVneo and JEG-3 cells (Figure 7(a-d)). The promoting effect of PTPRO knockdown on cell cycle progression was partially abolished by upregulation of ERp44.

### Downregulation of PTPRO facilitated the invasion of trophoblast cells by suppressing ERp44 expression

**Figure 8.**
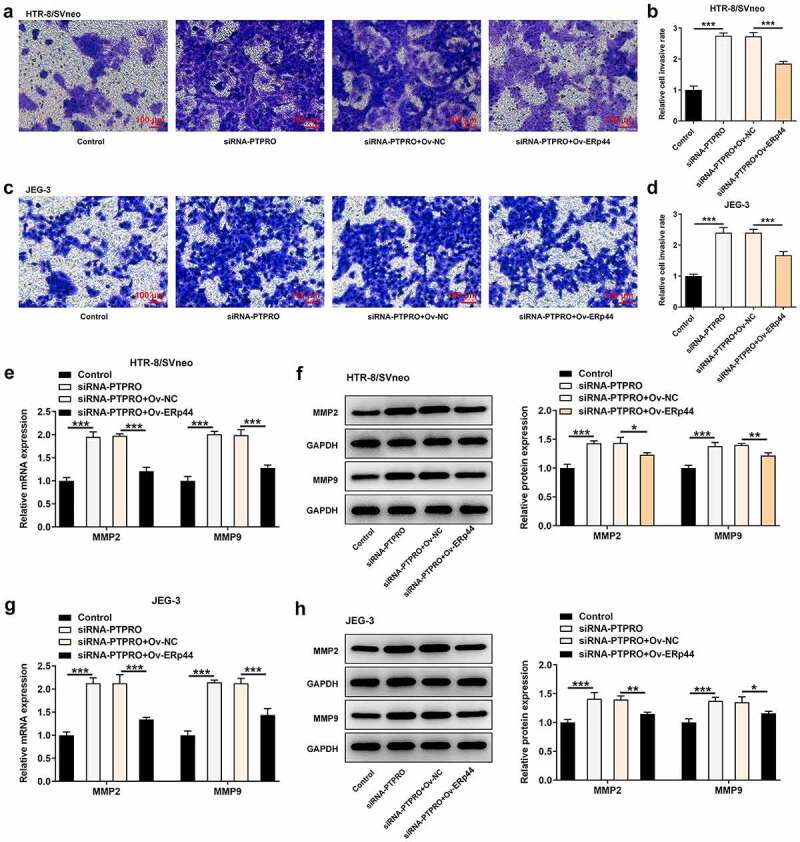
Downregulation of PTPRO facilitated the invasion of trophoblast cells by suppressing ERp44 expression. HTR-8/SVneo and JEG-3 cells were transfected with siRNA-PTPRO or co-transfected with siRNA-PTPRO and Ov-ERp44. (a, b) Transwell assay was performed to evaluate the invasion of HTR-8/SVneo cells. (c, d) Transwell assay was performed to evaluate the invasion of JEG-3 cells. (e) RT-qPCR was employed to detect the mRNA levels of MMP2 and MMP9 in HTR-8/SVneo cells. (f) Western blot analysis was employed to detect the protein levels of MMP2 and MMP9 in HTR-8/SVneo cells. (g) RT-qPCR was employed to detect the mRNA levels of MMP2 and MMP9 in JEG-3 cells. (h) Western blot analysis was employed to detect the protein levels of MMP2 and MMP9 in JEG-3 cells. * p < 0.05, **p < 0.01, ***p < 0.001

Here, transwell assay indicated that the enhancement of PTPRO knockdown on the invasive abilities of HTR-8/SVneo and JEG-3 cells was abrogated after ERp44 overexpression (Figure 8(a-d)). Additionally, reduced mRNA and protein levels of MMP2 and MMP9 in HTR-8/SVneo and JEG-3 cells caused by co-transfection with siRNA-PTPRO and Ov-ERp44 testified that elevation of ERp44 suppressed the invasion of trophoblast cells, reversing the promoting effect of PTPRO knockdown on the invasion of trophoblast cells (Figure 8(e-h)).

### Downregulation of PTPRO induced a stronger in vitro angiogenesis by suppressing ERp44 expression

**Figure 9.**
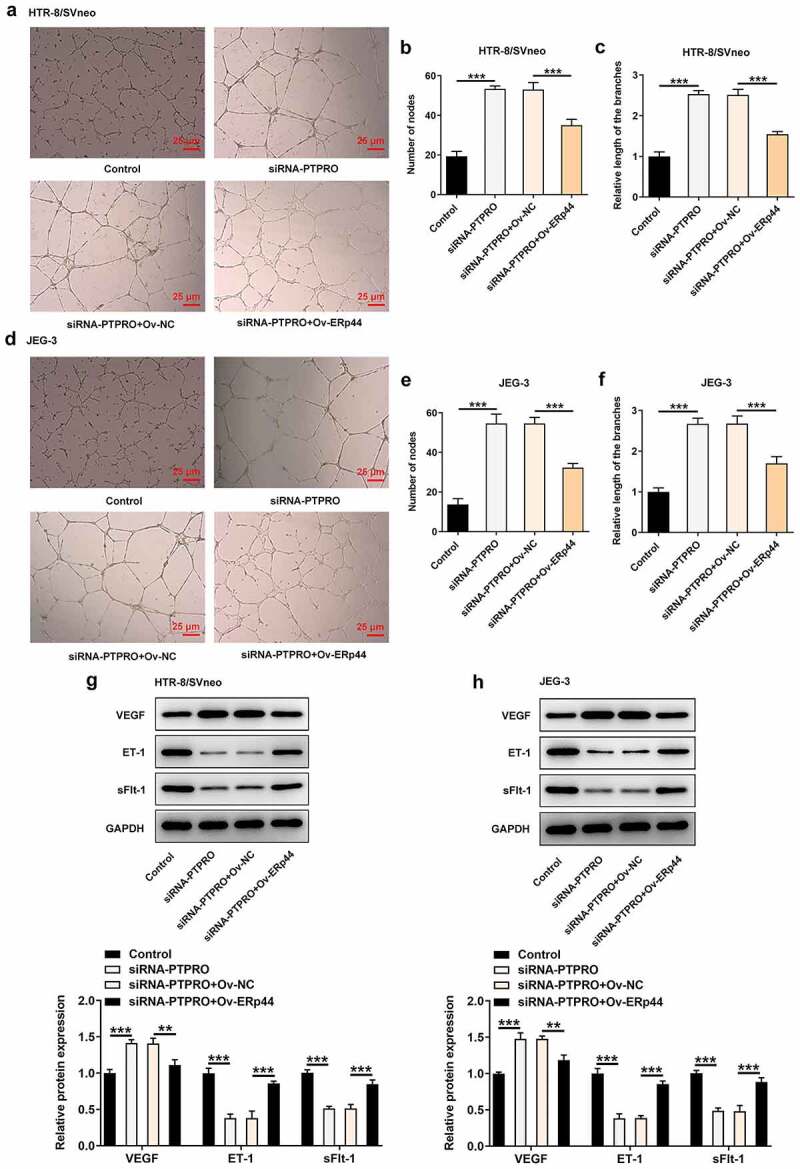
Downregulation of PTPRO induced a stronger in vitro angiogenesis by suppressing ERp44 expression. HTR-8/SVneo and JEG-3 cells were transfected with siRNA-PTPRO or co-transfected with siRNA-PTPRO and Ov-ERp44. HUVECs were incubated with the conditioned media (CM) of HTR-8/SVneo or JEG-3 cells. (a-c) The angiogenic activity of HUVECs incubated with the conditioned media (CM) of HTR-8/SVneo cells was tested using an in vitro tube formation assay. (d-f) The angiogenic activity of HUVECs incubated with the conditioned media (CM) of JEG-3 cells was tested using an in vitro tube formation assay. (g) Western blot analysis was employed to detect the protein levels of VEGF, ET-1 and sFlt-1 in HUVECs incubated with the conditioned media (CM) of HTR-8/SVneo cells. (h) Western blot analysis was employed to detect the protein levels of VEGF, ET-1 and sFlt-1 in HUVECs incubated with the conditioned media (CM) of JEG-3 cells. **p < 0.01, ***p < 0.001

Moreover, tube formation assay revealed that the stronger in vitro angiogenesis caused by PTPRO knockdown was repressed by ERp44 overexpression (Figure 9(a-f)). Besides, reduced expression of VEGF and elevated expressions of ET-1 and sFlt-1 in HTR-8/SVneo and JEG-3 cells caused by co-transfection with siRNA-PTPRO and Ov-ERp44 evidenced that elevation of ERp44 inhibited the angiogenic activity, abolishing the promoting effect of PTPRO knockdown on in vitro angiogenesis (Figure 9(g, h)).

## Discussion

The placenta is an important organ for material exchange between the fetus and the mother during pregnancy [16,17]. It is generally believed that PE belongs to placenta-derived diseases. As the derivatives of the trophectoderm layer, trophoblast cells participate in the endocrine, invasion and implantation processes of the placenta [18]. It is reported that PTPRO plays an important role in tumorigenesis by regulating cell proliferation, migration, invasion and inflammation [7]. Besides, PTPRO has been proved to be highly expressed in placenta-derived macrophages from patients with gestational diabetes mellitus and could participate in maintaining placental microenvironment balance [19]. Furthermore, literature has demonstrated that PTPRO expression is upregulated in placental mononuclear cells of patients with PE [9]. These findings pointed to the importance of PTPRO in pregnancy-related diseases including PE. In the current investigation, we evaluated the impact of PTPRO on the biological behaviors of trophoblast cells. Downregulation of PTPRO promoted the proliferation, cell cycle progression and invasion of HTR-8/SVneo and JEG-3 cells.

MMPs, a very important family of zinc-dependent enzymes, play a vital role in promoting cell invasion through various mechanisms such as degrading ECM and disrupting cell adhesion [20,21]. In our current research, it was verified that PTPRO knockdown elevated the expressions of MMP2 and MMP9 in HTR-8/SVneo and JEG-3 cells. In the theory of placental ischemia, abnormal recasting of maternal uterine spiral artery will further lead to placental insufficiency of blood supply and placental dysfunction, and eventually result in the manifestation of maternal diseases such as PE [22,23]. Therefore, regulating angiogenesis may be an important approach for PE therapies [24]. In this study, PTPRO knockdown markedly enhanced the angiogenic ability of HUVECs. VEGF is a key regulator of angiogenesis. sFlt1 is a potent antiangiogenic protein and ET-1 is a potent vasoconstrictor [25,26]. It has been testified by previous research that VEGF, sFlt1 and ET-1 can function to regulate angiogenesis of placental tissues during pregnancy [26,27]. In this current work, it was also confirmed that downregulation of PTPRO significantly increased the expression of VEGF and suppressed the expressions of sFlt1 and ET-1 in HUVECs. Downregulation of PTPRO induced a stronger in vitro angiogenesis.

Here, we presented evidence that PTPRO interacted with ERp44 in both HTR-8/SVneo and JEG-3 cells. Besides, downregulation of PTPRO reduced the expression of ERp44 in trophoblast cells, indicating that PTPRO might participate in the progression of PE by regulating ERp44 expression. Winship et al. have proved that interleukin-11 could trigger ER stress by stimulating ERp44, which may contribute to PE [28]. In addition, ERp44 is highly expressed in PE placentas and upregulation of miR-101 could inhibit trophoblast cell apoptosis by targeting ERp44 [14]. Furthermore, overexpression of ERp44 could negatively impact trophoblast cell proliferation and invasion and suppress angiogenesis [13]. In this present study, it was discovered that ERp44 overexpression suppressed the proliferation, cell cycle progression, invasion of trophoblast cells and inhibited in vitro angiogenesis, partially abolishing the biological functions of PTPRO knockdown.

## Conclusion

Taken together, this work investigated the specific functions of PTPRO in the biological behaviors of trophoblast cells and explored the underlying molecular mechanism. Functional experiments verified that downregulation of PTPRO facilitated the proliferation and invasion of trophoblast cells and induced a stronger in vitro angiogenesis by suppressing ERp44 expression. Findings above prompted that PTPRO/ERp44 might serve as important therapeutic targets for PE.

## Data Availability

The analyzed data sets generated during the present study are available from the corresponding author on reasonable request.
